# Supplementary figures and images for: Prognostic value of plasma pentraxin 3 levels in patients with septic shock admitted to intensive care
Source: PLoS One. 2020 Dec 10;15(12):e0243849. doi: 10.1371/journal.pone.0243849 (PMC7728227; doi:10.1371/journal.pone.0243849)

S1 Fig. ROC Curves for Severity Scores and biomarkers with respect to ‘in hospital mortality’.


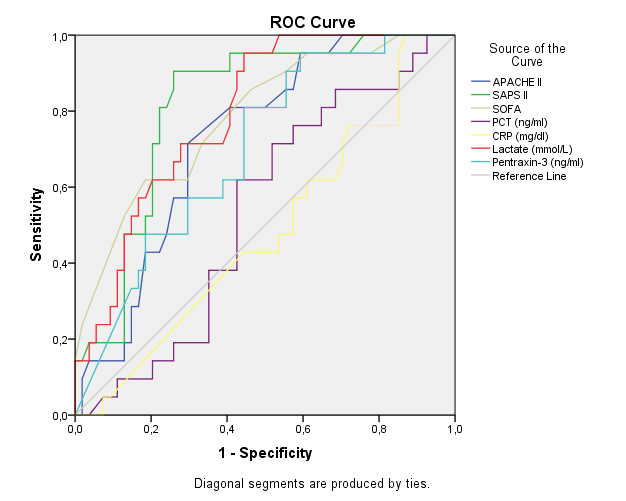

Supplement: S1 Fig — (DOC) [file pone.0243849.s001.doc]
