# Supplementary material for: Prognostic value of plasma pentraxin 3 levels in patients with septic shock admitted to intensive care
Source: PLoS One. 2020 Dec 10;15(12):e0243849. doi: 10.1371/journal.pone.0243849 (PMC7728227; doi:10.1371/journal.pone.0243849)
Supplement: Attachment — Submitted filename: RESPOND TO REVIEWERS.docx [file pone.0243849.s003.docx]

RESPOND TO REVIEWERS

Attached to this email I am sending you a new manuscript that includes all the changes suggested by the reviewers. In addition, English has been reviewed by a professional translator specialized in scientific articles.

- The language used in the revised sections of the manuscript needs to be improved. In particular:

1. I suggest using an alternative phrase for describing/comparing the magnitude of AUC. Consider using "lower" instead of "smaller".

We have made the suggested change.

2. Please consider rephrasing the Conclusion sentence of the Abstract: instead of "Our results support the prognostic value", consider the following: "Our results suggest that the prognostic value of... may support..."

We have made the suggested change

3. Please rephrase the following: "But even they have limited abilities..."

We have made the suggested change

4. Please, try rephrasing the following sentence: "Lactate was the biomarker with higher associations..."

We have made the suggested change

5. "Our results also support the severity scores..." Please consider using "Our results also suggest that..."

We have made the suggested change

6. Please consider rephrasing: "slight increase"

We have made the suggested change

7. Please revise the following sentence: "These conflicting outcomes may due to the heterogeneity..."

We have made the suggested change
